# Magnetic Seizure Therapy Compared to Electroconvulsive Therapy for Schizophrenia: A Randomized Controlled Trial

**DOI:** 10.3389/fpsyt.2021.770647

**Published:** 2021-11-25

**Authors:** Jiangling Jiang, Jin Li, Yuanhong Xu, Bin Zhang, Jianhua Sheng, Dengtang Liu, Wenzheng Wang, Fuzhong Yang, Xiaoyun Guo, Qingwei Li, Tianhong Zhang, Yingying Tang, Yuping Jia, Zafiris J. Daskalakis, Jijun Wang, Chunbo Li

**Affiliations:** ^1^Shanghai Key Laboratory of Psychotic Disorders, Shanghai Mental Health Center, Shanghai Jiao Tong University School of Medicine, Shanghai, China; ^2^Psychological and Psychiatric Neuroimage Lab, The Affiliated Brain Hospital of Guangzhou Medical University, Guangzhou, China; ^3^Department of Psychiatry, Tongji Hospital of Tongji University, Shanghai, China; ^4^Department of Psychiatry, University of California, San Diego, La Jolla, CA, United States; ^5^Institute of Psychology and Behavioral Science, Shanghai Jiao Tong University, Shanghai, China; ^6^Center for Excellence in Brain Science and Intelligence Technology (CEBSIT), Chinese Academy of Science, Shanghai, China; ^7^Brain Science and Technology Research Center, Shanghai Jiao Tong University, Shanghai, China

**Keywords:** magnetic seizure therapy, electroconvulsive therapy, schizophrenia, randomized controlled trial, cognitive functions

## Abstract

**Background:** Magnetic seizure therapy (MST) is a potential alternative to electroconvulsive therapy (ECT). However, reports on the use of MST for patients with schizophrenia, particularly in developing countries, which is a main indication for ECT, are limited.

**Methods:** From February 2017 to July 2018, 79 inpatients who met the DSM-5 criteria for schizophrenia were randomized to receive 10 sessions of MST (43 inpatients) or ECT (36 inpatients) over the course of 4 weeks. At baseline and 4-week follow-up, the Positive and Negative Syndrome Scale (PANSS) and the Repeatable Battery for the Assessment of Neuropsychological Status (RBANS) were used to assess symptom severity and cognitive functions, respectively.

**Results:** Seventy-one patients who completed at least half of the treatment protocol were included in the per-protocol analysis. MST generated a non-significant larger antipsychotic effect in terms of a reduction in PANSS total score [*g* = 0.17, 95% confidence interval (CI) = −0.30, 0.63] and response rate [relative risk (RR) = 1.41, 95% CI = 0.83–2.39]. Twenty-four participants failed to complete the cognitive assessment as a result of severe psychotic symptoms. MST showed significant less cognitive impairment over ECT in terms of immediate memory (*g* = 1.26, 95% CI = 0.63–1.89), language function (*g* =1.14, 95% CI = 0.52–1.76), delayed memory (*g* = 0.75, 95% CI = 0.16–1.35), and global cognitive function (*g* = 1.07, 95% CI = 0.45–1.68). The intention-to-treat analysis generated similar results except for the differences in delayed memory became statistically insignificant. Better baseline cognitive performance predicted MST and ECT response.

**Conclusions:** Compared to bitemporal ECT with brief pulses and age-dose method, MST had similar antipsychotic efficacy with fewer cognitive impairments, indicating that MST is a promising alternative to ECT as an add-on treatment for schizophrenia.

**Clinical Trial Registration:** ClinicalTrials.gov, identifier: NCT02746965.

## Introduction

Schizophrenia is generally characterized by marked dysfunction in cognition, behavior, and emotion. Patients with schizophrenia suffer from pronounced functional impairment (1), as well as considerable disability (2). Moreover, the economic burden of the disease can be up to 1% of the gross domestic product of a country (3). While antipsychotics are the mainstay treatment for schizophrenia, ~30% of patients do not respond fully to pharmacotherapy (4).

Electroconvulsive therapy (ECT) is an important treatment option when the response to pharmacotherapy alone is unsatisfactory, or when rapid improvement in global functioning and psychotic symptoms is desired (5). Eighty years have passed since its development, but clinicians still use ECT to treat patients with severe mental disorders (6). However, cognitive side effects, such as amnesia, headache, and disorientation, are common (7). The substantial impedance of the scalp and skull leads to widespread electrical stimulation through the whole brain, which is thought to underlie the cognitive side effects of ECT (8).

Owing to their ability to pass through the scalp and skull without resistance, magnetic pulses can generate considerable focal stimulation in the brain. Consequently, researchers have made great efforts to replace electric currents with magnetic pulses in the deliberate induction of therapeutic seizures. Magnetic seizure therapy (MST) was first successfully administered in humans in the early 2000s (9). Compared to any form of ECT, MST delivers energy to more superficial cortical regions, avoiding the stimulation of the cognition-related sub-cortex, such as the hippocampus and basal ganglia (8).

The safety of MST has been well documented in both animal experiments (10, 11) and human studies (12). When compared to ECT, MST has shown comparable efficacy and a superior cognitive profile in patients with depression (12). Although ECT is widely administered to patients with schizophrenia, especially in Asia and East Europe, the effects of MST on this population have not yet been well studied. Recently, two open-label trials preliminarily demonstrated the feasibility of MST in treating schizophrenia (13, 14). However, randomized controlled trials (RCTs) have yet to be conducted.

The primary aim of this study was to investigate the clinical and cognitive effects of MST in patients with schizophrenia. We hypothesized that the efficacy of MST is comparable to that of ECT and that the cognitive side effects of MST are less severe. Our secondary aim was to explore the demographic and clinical predictors of response to MST and ECT.

## Methods

### Participants and Study Design

From February 2017 to July 2018, inpatients from Shanghai Mental Health Center in China were recruited into this double-blind, parallel RCT of MST and ECT (clinicaltrials.gov registration number: NCT02746965). The calculation of sample size was descripted in Supplementary Materials. However, this RCT has been suspended because the coils were worn out. The authors assert that all procedures contributing to this work comply with the ethical standards of the relevant national and institutional committees on human experimentation and with the Helsinki Declaration of 1975, as revised in 2008 (15). The Institutional Review Board of the Shanghai Mental Health Center approved this protocol (2014-30R). Recruitment methods included the introduction of clinical personnel and advertisements posted in the wards. Patients who were interested in participating in this study signed informed consent after being screened according to the study criteria.

The inclusion criteria were as follows: (1) 18–55 years old; (2) Diagnostic and Statistical Manual of Mental Disorders, Fifth Edition (DSM-5) diagnosis of schizophrenia; (3) clinically indicated convulsive therapy, including for the treatment of severe psychomotor excitement or retardation, suicide attempts, highly aggressive behavior, pharmacotherapy intolerance, and ineffectiveness of antipsychotics (total or partial lack of response to previous treatment using at least one antipsychotic at adequate doses and periods), as assessed by two attending doctors; (4) Positive and Negative Syndrome Scale (PANSS) (16) score ≥ 60; and (5) patients who provided written informed consent for participating in the study and publication of this case series.

The exclusion criteria were as follows: (1) diagnosis of other mental disorders; (2) severe physical disease, such as stroke, heart failure, liver failure, neoplasm, or immune deficiency; (3) laboratory abnormality that could impact the treatment efficacy or the participants' safety; (4) failure to respond to an adequate trial of ECT; (5) pregnancy or intention to become pregnant during the study; and (6) other conditions that investigators considered inappropriate for participation in this trial (e.g., participating in other clinical trials).

A random sequence of allocation with a ratio of 1:1 was generated using SAS 9.3 (SAS Institute Inc., USA) by an independent biostatistician who had no access to information on the study subjects. Each subject received a number within a concealed opaque envelope indicating their randomization assignment. The treatment code was provided to the treating clinician following the baseline assessment, but prior to the first treatment session. All procedures prior to treatment and the room setup were made identical to ensure the blinding of patients (e.g., presence of both ECT and MST equipment). Clinical and cognitive assessments were conducted by a trained psychiatrist who was blinded to the treatment group.

### MST and ECT Procedure

Generally, the setting resembled that of ECT clinical practice in China (17). In addition to treatment as usual (TAU), the participants were supposed to receive ten sessions of MST/ECT over 4 weeks, with three sessions per week during the first 2 weeks, and two sessions per week during the following 2 weeks. The MST/ECT was administered under general anesthesia with intravenous etomidate (0.21–0.3 mg/kg) and propofol (1.82–2.44 mg/kg). Intravenous succinylcholine (1 mg/kg) was used as a muscle relaxant, and intravenous atropine (0.5 mg) was used to reduce airway secretions.

MST was administered with a MagPro MST (MagVenture A/S, Denmark) at 50 Hz and 100% output. The pulse width was 370 μs, and the peak intensity of the magnetic field was 4.2 Tesla. A titration method was employed to determine the duration of the magnetic stimulation; the duration began at 4 s and was increased by 4 s in each subsequent session up to a maximum of 20 s (i.e., 200–1,000 pulses per session). If the seizure quality was poor (seizure duration <15 s) in a certain session, the increment of the stimulation duration was 8 s during the next session. If no seizures were generated, an extra stimulation lasting for 20 s was administered immediately. For depression, there is evidence of better seizure quality (18) and therapeutic effect (19) when MST is administered with pulse frequencies of 25 and 50 Hz rather than 100 Hz. Moreover, the results of our pilot study showed that 25 Hz pulses may not be optimal in the studied population of Chinese patients with schizophrenia (14). In addition, the effectiveness of the titration method in generating seizure activity among patients with schizophrenia has been demonstrated in previous studies (13, 14). Magnetic stimulation was delivered via a twin coil (Twin Coil—XS; MagVenture A/S, Denmark) with its midline on the vertex. The details of the coil replacement were stated in our previous article (14).

Bitemporal ECT was administered using the Thymatron System IV device (Somatics, USA). The pulse width of the electrical stimulus was set to 0.5 ms. The energy used in the first session was set according to patient's age, and the percent energy used in the following sessions was increased by 5%. If the seizure was inadequate (seizure duration <25 s), the maximum dosage was administered in the subsequent session. If no seizures were induced, the maximum dosage was administered immediately. The Thymatron IV device with left and right frontal leads was also utilized to record the electroencephalogram (EEG) during MST and ECT.

### Assessments

At baseline and at the completion of all the treatments, the PANSS and the Repeatable Battery for the Assessment of Neuropsychological Status (RBANS, Form A at baseline and Form B at the end-point) (20) were employed to measure the improvements in the psychotic symptoms (primary outcome) and cognitive effects, respectively. The RBANS consists of 12 subtests that form five age-adjusted index scores, including immediate memory, visuospatial function, language, attention, and delayed memory. The RBANS has shown good reliability and validity in Chinese patients with schizophrenia (21). It measures delayed memory, which is selective impaired following ECT (22). In fact, ECT improves all domains of the MATRICS Consensus Cognitive Battery (MCCB) (23). Besides, in our pilot study, we found RBANS more feasible than MCCB for our targeted population, i.e., inpatients with severe psychosis (14). As a consequence, we employed RBANS instead of MCCB. The response rate was defined as a ≥ 25% reduction in the total PANSS score (24). A delayed memory deficit was defined as a ≥ 10 % reduction in the RBANS delayed memory score (25).

### Statistical Analyses

R version 3.5.1 (https://www.r-project.org) was used to perform the statistical analyses. Chi-square test, independent t-test, and Mann–Whitney U test were used to compare the demographic and clinical characteristics between the two intervention groups according to the measurement categories (dichotomous variables, continuous variables with normal distribution, and continuous variables with skewed distribution, respectively). The Kolmogorov–Smirnov test was used to differentiate skewed distributions from normal distributions. When a cell in the four-fold table had an expected count of <5, Fisher's exact test was used instead of the chi-square test. The dosage of different antipsychotic agents was standardized using the defined daily dose (DDD) approach (https://www.whocc.no/atc_ddd_index/).

Full factorial linear mixed effect models (LMEs) with repeated measures were utilized to investigate the within-group time effect (baseline/post-treatment) and the between-group time × group (MST/ECT) interaction on psychotic symptoms and cognitive functions, with the individual differences as a random effect of slope and intercept, and the antipsychotic dosage as a covariate. For measures with significant time × interactions, within-group LMEs were performed with correction for multiple comparisons using the Bonferroni method. Hedge's *g* with its 95% CI was calculated as a measure of effect size for continuous variables, while relative risk (RR) with 95% CI was calculated for dichotomous variables. Intention-to-treat (ITT) analyses were performed to test the robustness of per-protocol analyses. The worst-case scenarios was used to impute missing data.

In addition, logistic regression with baseline levels and antipsychotic dosage as covariates was employed to compare the rate of clinical response and delayed memory deficit between ECT and MST, and to explore the potential predictors. Moreover, each significant predictor was put into a multivariate logistic regression model in a stepwise manner, with antipsychotic dosage as a covariate. To estimate the accuracy of the prediction model, we plotted the smoothed receiver operating characteristic (ROC) curve and calculated the area under the curve (AUC) using the bootstrap approach with an iteration of 10,000.

## Results

### Participants

**Figure 1:**
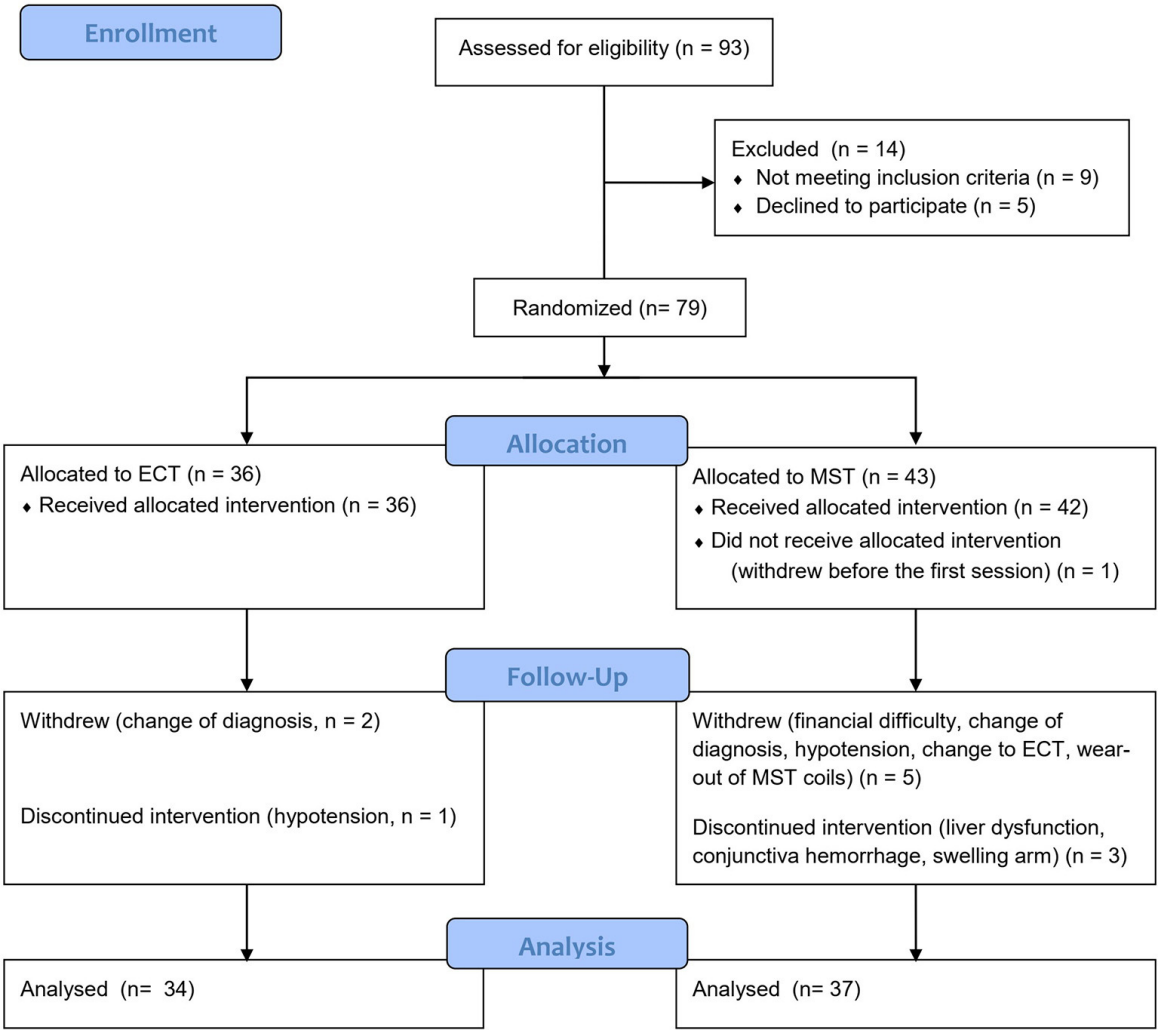
Study flow chart of the ongoing randomized controlled study. MST, magnetic seizure therapy; ECT, electroconvulsive therapy.

Of the 93 screened patients, 79 were eventually recruited and randomized. Because we used a simple randomized design, the number of participants in the two groups was not equal in this interim analysis (36 patients in the ECT group and 43 the MST group). Six subjects were excluded from the final analysis for the following reasons: one patient in the MST group withdrew prior to the first treatment due to financial difficulty; another patient withdrew due to financial difficulty after the second session; one patient withdrew because a tumor was found in his brain after the fifth session; one patient withdrew after the first session due to hypotension (80/50 mmHg); one patient in the MST group, who felt that his symptoms did not improve after three sessions of treatment, predicted that his allocation was MST, and requested withdrawal from the study to receive ECT; one patient withdrew due to wearing of the MST coils after the second session. Moreover, two patients in the ECT group withdrew after the third session due to a change in diagnosis to brain tumor in one patient and multiple sclerosis, in the other; these patients were also excluded from the analysis. Figure 1 shows the details of the study flow. The rate of completion of the ten sessions did not differ significantly between ECT and MST (*p* of Fisher's exact test = 0.208).

Of the 71 subjects included in the per-protocol analysis, three patients discontinued MST after the eighth session for the following reasons: liver dysfunction (the level of glutamic oxaloacetic transaminase and glutamic-pyruvic transaminase reached twice the upper limit of normal range); conjunctiva hemorrhage; and swelling in the right arm. In addition, one patient discontinued ECT after the eighth session due to hypotension (80/50 mmHg). All four patients recovered from adverse events, and none of them had a prolonged hospital stay (Figure 1).

All of the patients included in the analysis were taking atypical antipsychotics, 11 of who took benzodiazepines, none of who took antiepileptics, and 16 of who were clozapine-resistant. None of our participants had a comorbid diagnosis of substance abuse or depression. Twenty-four patients failed to receive the RBANS at least once due to marked auditory hallucinations or functional impairments related to psychotic symptoms. The demographic characteristics were balanced between the two treatment groups (Table 1). However, at baseline, the ECT group had significantly higher general psychopathology scores and total PANSS scores, with better immediate memory at a trend level, than the MST group. The time from the last treatment session until the cognitive assessment was 4.3 ± 3.5 days for MST, and 3.0 ± 1.8 days for ECT (*t* = −1.6, *p* = 0.119).

**Table 1:** Baseline demographic, clinical, and cognitive characteristics of participants.

	**MST**	**ECT**	*****χ^2^/t/Z*****	***p***
Gender (male:female)	19:24	14:22	0.23^a^	0.634
Age (year)	31.3 ± 9.3	33.8 ± 10.8	1.14^b^	0.257
Education year	13.0 ± 2.9	12.2 ± 3.7	1.07 ^b^	0.286
Being married (yes:no)	10:33	11:25	0.54^a^	0.465
Being employed (yes:no)	11:32	14:22	1.60^a^	0.205
Smoke (yes:no)	7:36	2:34	/^d^	0.170
Onset age (year)	22.5 ± 7.6	25.7 ± 9.8	1.25^c^	0.210
Disease duration (year)	8.0 ± 7.0	7.8 ± 6.8	0.11 ^b^	0.911
Episode	3.9 ± 2.0	3.8 ± 2.4	0.20^b^	0.845
DDD	1.45 ± 0.89	1.53 ± 0.66	0.46^b^	0.648
Clozapine-resistant (yes:no)	11:32	8:28	0.12^a^	0.732
Benzodiazepines	8:35	3:33	/^d^	0.328
PANSS				
Positive symptom	26.6 ± 4.6	28.1 ± 4.6	1.44^b^	0.153
Negative symptom	21.0 ± 6.9	22.0 ± 6.0	0.68^b^	0.496
General psychopathology	44.6 ± 8.4	48.9 ± 6.8	2.45^b^	0.016*
Total score	92.3 ± 13.0	99.1 ± 11.6	2.43^b^	0.018*
RBANS				
Received at least one time (yes:no)	31:12	24:12	0.273^a^	0.601
Immediate memory	68.5 ± 16.3	78.5 ± 22.5	1.91^b^	0.062
Visuospatial function	85.3 ± 18.6	86.6 ± 16.4	0.28^b^	0.784
Language	81.3 ± 16.3	79.9 ± 18.6	0.28^b^	0.777
Attention	90.7 ± 14.8	93.7 ± 14.0	0.77^b^	0.444
Delayed memory	71.8 ± 20.4	75.5 ± 23.2	0.57^c^	0.568
Total index	74.2 ± 15.0	77.7 ± 18.7	0.80^b^	0.439

*p < 0.05.

*^a^X^2^, ^b^t, ^c^Z, ^d^Fisher's exact test.*

*MST, magnetic seizure therapy; ECT, electroconvulsive therapy; DDD, defined daily dose; PANSS, Positive and Negative Syndrome Scale; RBANS, Repeatable Battery for the Assessment of Neuropsychological Status*.

### Clinical Outcomes

**Figure 2:**
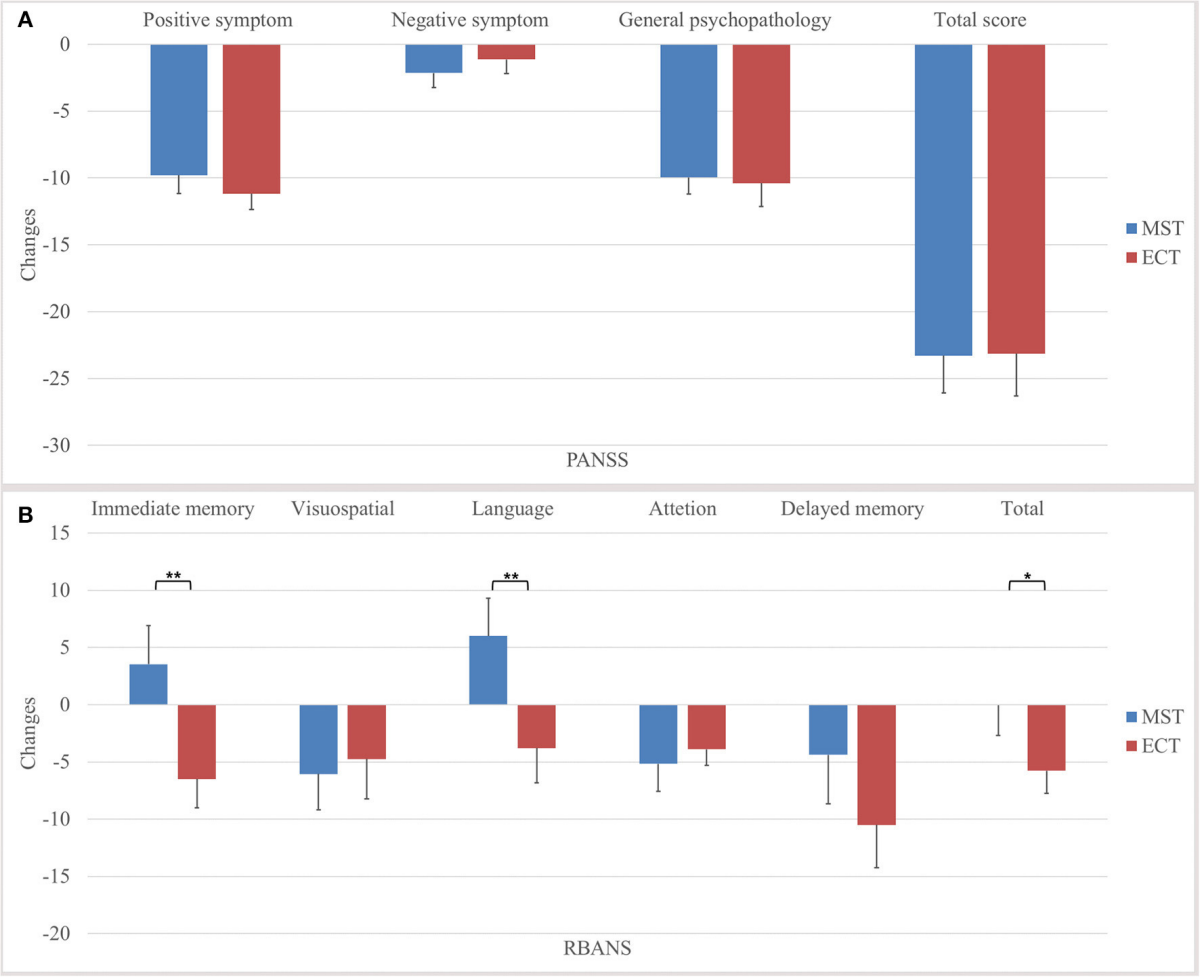
Changes in psychotic symptoms and cognitive functions in the intention-to-treat dataset. We found no significant differences between MST and ECT in the **(A)** changes of total score and subscale scores of PANSS, but significant differences were found in the **(B)** changes of immediate memory, language, delayed memory, and total index of RBANS. *p ≤ 0.05, **p ≤ 0.01. PANSS, positive and negative syndrome Scale, RBANS, repeatable battery for the assessment of neuropsychological status, MST, magnetic seizure therapy, ECT, electroconvulsive therapy.

For primary outcomes, both seizure therapies significantly reduced the psychotic symptoms, in terms of the positive symptoms and general psychopathology, with large effect sizes, in the ITT analysis (Table 2, Figure 2). Direct comparisons between ECT and MST did not reveal any significant changes in psychotic symptoms. In the per-protocol analysis, the patterns of psychotic symptom improvement were consistent with those in the ITT analysis (Table 3).

**Table 2:** The effects of MST and ECT on psychotic symptoms and cognitive functions in the intention-to-treat dataset.

	**Group**	**Changes**	**Time**			**Time** **×** **Group**		
			***t***	***p***	***g* (95% CI)**	***t***	***p***	***g* (95% CI)**
**PANSS**								
Positive score	MST	−9.8 ± 8.3	−8.65	0.000***	−1.96 (−2.50 to −1.42)	−0.79	0.434	−0.18 (−0.62 to 0.26)
	ECT	−11.2 ± 7.0						
Negative score	MST	−2.1 ± 6.7	−1.05	0.296	−0.24 (−0.68 to 0.21)	−0.68	0.496	−0.15 (−0.60 to 0.29)
	ECT	−1.1 ± 6.2						
General psychopathology	MST	−10.0 ± 7.5	−7.11	0.000***	−1.61 (−2.12 to −1.10)	0.22	0.827	0.05 (−0.39 to 0.49)
	ECT	−10.4 ± 10.1						
Total score	MST	−23.3 ± 16.9	−7.86	0.000***	−1.78 (−2.31 to −1.26)	−0.04	0.97	−0.01 (−0.45 to 0.43)
	ECT	−23.1 ± 18.5						
**RBANS**								
Immediate memory	MST	3.5 ± 16.8	−2.78	0.008*	−0.76 (−1.31 to −0.21)	3.14	0.003**	0.86 (0.30–1.41)
	ECT	−6.5 ± 11.7						
Visuospatial function	MST	−6.0 ± 15.7	−1.87	0.068	−0.51 (−1.05 to 0.03)	−0.25	0.805	−0.07 (−0.60 to 0.46)
	ECT	−4.8 ± 16.2						
Language	MST	6.0 ± 16.5	−1.51	0.136	−0.41 (−0.95 to 0.13)	2.81	0.007**	0.77 (0.21–1.32)
	ECT	−3.8 ± 14.4						
Attention	MST	−5.2 ± 12.0	−2.51	0.015*	−0.69 (−1.23 to −0.14)	−0.43	0.670	−0.12 (−0.65 to 0.42)
	ECT	−3.9 ± 6.7						
Delayed memory	MST	−4.4 ± 21.4	−3.38	0.001**	−0.92 (−1.48 to −0.36)	1.56	0.124	0.43 (−0.11 to 0.96)
	ECT	−10.5 ± 17.5						
Total index	MST	0.0 ± 13.3	−3.13	0.003**	−0.85 (−1.41 to −0.30)	2.33	0.024*	0.64 (0.09–1.18)
	ECT	−5.8 + 9.3						

*p ≤ 0.05; **p ≤ 0.01; ***p ≤ 0.001. CI, confidence interval; ECT, electroconvulsive therapy; MST, magnetic seizure therapy; PANSS, the Positive and Negative Syndrome Scale; RBANS, the Repeatable Battery for the Assessment of Neuropsychological Status.

**Table 3:** The effects of MST and ECT on psychotic symptoms and cognitive functions in the per protocol dataset.

	**Group**	**Changes**	**Time**			**Time** **×** **Group**		
			***t***	***p***	***g* (95% CI)**	***t***	***p***	***g* (95% CI)**
**PANSS**								
Positive score	MST	−12.2 ± 6.1	−11.08	0.000***	−2.64 (−3.28 to −2.00)	−0.17	0.869	−0.04 (−0.51 to 0.43)
	ECT	−11.9 ± 6.4						
Negative score	MST	−3.8 ± 5.7	−1.83	0.072	−0.44 (– 0.91 to 0.04)	−1.46	0.148	−0.35 (−0.82 to 0.12)
	ECT	−1.8 ± 5.7						
General psychopathology	MST	−11.9 ± 6.1	−8.97	0.000***	−2.14 (−2.73 to −1.55)	−0.14	0.892	−0.03 (−0.50 to 0.43)
	ECT	−11.6 ± 8.9						
Total score	MST	−27.9 ± 13.4	−9.91	0.000***	−2.36 (−2.97 to −1.75)	−0.70	0.487	−0.17 (−0.63 to 0.30)
	ECT	−25.4 ± 16.4						
**RBANS**								
Immediate memory	MST	10.9 ± 17.1	−2.38	0.021*	−0.70 (−1.29 to −0.11)	4.29	0.000***	1.26 (0.63–1.89)
	ECT	−7.4 ± 11.0						
Visuospatial function	MST	−2.7 ± 15.3	−1.09	0.281	−0.32 (−0.90 to −0.26)	0.21	0.836	0.06 (−0.51 to 0.63)
	ECT	−3.7 ± 16.4						
Language	MST	14.6 ± 15.4	−0.95	0.346	−0.28 (−0.86 to −0.30)	3.89	0.000***	1.14 (0.52–1.76)
	ECT	−3.2 ± 16.0						
Attention	MST	−2.4 ± 10.5	−2.56	0.014*	−0.75 (−1.35 to −0.16)	0.97	0.336	0.29 (−0.29 to 0.86)
	ECT	−5.0 ± 7.3						
Delayed memory	MST	1.8 ± 21.2	−3.08	0.004**	−0.91 (−1.51 to −0.30)	2.57	0.014*	0.75 (0.16–1.35)
	ECT	−12.9 ± 17.7						
Total index	MST	5.0 ± 13.1	−2.97	0.005**	−0.87 (−1.47 to −0.27)	3.63	0.001***	1.07 (0.45–1.68)
	ECT	−7.4 ± 9.6						

*p ≤ 0.05; **p ≤ 0.01; ***p ≤ 0.001. CI, confidence interval; ECT, electroconvulsive therapy; MST, magnetic seizure therapy; PANSS, the Positive and Negative Syndrome Scale; RBANS, the Repeatable Battery for the Assessment of Neuropsychological Status.

The response rates for MST and ECT were comparable (55.8 vs. 50.0%, *Z* = 0.84, *p* = 0.401, RR = 1.14, 95% CI = 0.72–1.80) in the ITT analysis. The per-protocol analysis also generated comparable response rates for MST and ECT (64.9 vs. 52.9 %, *Z* = 1.13, *p* = 0.258, RR = 1.41, 95% CI = 0.83–2.39).

### Cognitive Outcomes

Time × group interactions were found among immediate memory, language function, and the total index of RBANS, with medium to large effect sizes (Table 2, Figure 2) in the ITT analysis. In the per-protocol analysis, the patterns of cognitive changes were consistent with those in the ITT analysis (Table 3) except for the presence of time × group interaction in delayed memory. The within-group analysis is detailed in the Supplementary Material.

The rate of delayed memory deficit was lower in the MST group than in the ECT group (41.9 vs. 6.67%, *Z* = −1.741, *p* = 0.082, RR = 0.63, 95% CI = 0.27–1.48) at a trend level in the ITT analysis. The per-protocol analysis also generated rates of delayed memory deficit in favor of MST (28.0 vs. 63.6%, *Z* = −2.28, *p* = 0.023, RR = 0.27, 95% CI = 0.09–0.86).

### Predictors of Outcomes

A short duration of disease (*Z* = 2.18, *p* = 0.029), non-clozapine resistance (*Z* = 2.30, *p* = 0.022), and better baseline attention (*Z* = 1.96, *p* = 0.050) predicted MST response. Duration of disease and attention remained in the stepwise multivariate model, with the best sensitivity of 87.5% and specificity of 100% under the threshold of 0.869, and with an AUC of 95.9% (95 % CI = 88.9–99.9%; Supplementary Figure 1A1). In contrast, higher education years (*Z* = 2.30, *p* = 0.021) and better baseline cognitive function, including immediate memory (*Z* = 2.23, *p* = 0.026), attention (*Z* = 2.18, *p* = 0.029), delayed memory (*Z* = 2.11, *p* = 0.035), and global cognitive function (*Z* = 2.17, *p* = 0.030), predicted ECT responses. When these factors were included in the multivariate model, only immediate memory was selected using the stepwise approach, with the best sensitivity of 84.6% and specificity of 77.8% under the threshold of 0.547, and an AUC of 88.7% (95% CI = 72.9–97.6%; Supplementary Figure 1B1). Nevertheless, no baseline demographic, clinical, or cognitive factors predicted delayed memory deficits in the ECT or MST groups.

## Discussion

To the best of our knowledge, this is the first study to compare the efficacy and cognitive side effects of MST and ECT in patients with schizophrenia. Add-on MST was generally safe among this population and was effective in treating psychotic symptoms with limited cognitive impairment. The clinical response pattern did not differ significantly between MST and ECT, in that the lower bound of the 95% CI of the effect size of total reduction in PANSS score for MST was larger than the mean estimate for ECT. However, we also found that ECT produces much larger short-term cognitive impairment than MST in immediate memory, language function, and the total index of the RBANS, and maybe delayed memory as well. Combining these results, the results of the present study suggest that MST is a promising alternative treatment to ECT for the treatment of schizophrenia, with a comparable antipsychotic effect and less cognitive impairments to that of bitemporal ECT with brief pulses and age-dose method in short term. In addition, we identified several potential predictors of clinical response following seizure therapies; among which, baseline immediate memory was the most predictive of ECT, and the duration of disease and baseline attention were most predictive of MST.

Similar to the RCTs comparing MST and ECT in patients with depression (26, 27), symptom improvement did not differ significantly between patients who received MST and those who received ECT. It should be noted that the response rate of MST was ~5–10 % higher than that of ECT, but this difference was not significant. The imbalance in baseline symptom severity might have contributed to this difference. In a study conducted by Zhang et al. (28), the response rates of ECT in Chinese patients with schizophrenia have been reported to be more than 70%. It is noted that their study focused on adolescent patients who were more likely to benefit from ECT (29).

The present study revealed the less cognitive impairments following MST over ECT for patients with schizophrenia, confirming the findings from previous trial in patients with depression (26, 27, 30). These findings are also supported by parametric modeling studies (31) and animal experiments (11), in which ECT delivered a large amount of energy into the subcortical area with the accumulation of neuroglial cells in the hippocampus, while MST barely affected this neurocognition-related structure. In addition, MST and ECT modulate human electrophysiological activity differently in terms of the EEG complexity, which is associated with the cognitive outcome, providing *in vivo* insight into the cognitive superiority of MST (32). The effects of ECT on cognitive function are domain-dependent and not necessarily harmful. Indeed, ECT improves most cognitive domains at more than 3 days post-treatment (22). Similarly, we found that MST also improved some domains of cognitive function. As the cognitive side effects will be gradually resolved 2 weeks after the end of ECT (22), the less cognitive impairments following MST in cognitive performance needs further confirmation by future studies with longer follow-up periods. Moreover, a recent open-label self-control trial found a decline in autobiographic memory following MST (13), which remains to be confirmed by RCT with masked assessment.

With the exception of cognitive side effects, ECT has become a much safer physical therapy following the introduction of general anesthesia (33, 34). Consistent with previous depression studies (12), the present study found no serious adverse events among patients with schizophrenia who received MST. Propofol can inhibit cytochrome P450 (35) and affect hepatocellular integrity (36) therefore, it might be associated with liver injury after seizure therapies (37). In addition, the hypotension found in patients receiving ECT and MST may be another side effect of propofol, which reduces blood pressure by increasing the release of nitric oxide (38) and inhibiting baroreceptors (39), while seizure therapies act to raise it (40). Furthermore, the peripheral levels of norepinephrine, epinephrine, adrenocorticotrophic hormone, and arginine vasopressin are elevated during and after seizure therapies (41); these factors are responsible for blood vessel constriction, and thus underlie the side effects of hypertension and bleeding conjunctiva. In the present study, the history of conjunctival hemorrhage was not reported by the patient until it occurred again after the eighth session of MST, indicating that a detailed history and close ophthalmologic examination are needed for patients at high risk before seizure therapies. Thromboembolism might be the cause of the swelling of the arm of the patient receiving MST in the present study. However, seizure therapies do not increase the risk of thromboembolism (42, 43). On the other hand, this patient took paliperidone, which has a profile of thromboembolic side effects (44). In short, the medication confounder made it impossible to determine whether most of the adverse events were seizure-therapy-related.

Baseline cognitive function could predict the treatment response to both MST and ECT. Higher cognitive functions demand relative preservation of neural structure and function (45, 46), which were also predictive of treatment response of ECT (47, 48). However, such preservation is progressively damaged with the increase in disease duration (49). As a consequence, a shorter duration of disease is associated with better response to ECT for schizophrenia (50) and MST for depression (51). Nevertheless, we only found an association between treatment response and disease duration and clozapine resistance among patients who received MST, not ECT, possibly due to the small sample size.

The present study was mainly limited by the small sample size due to coil malfunction, which prohibited us from performing non-inferior analysis, hence reducing the power of concluding that MST and ECT have comparable antipsychotic efficacy. The small sample size may also be the cause of unbalanced baseline severity of psychotic symptoms and immediate memory, which also reduces the certainty that MST has similar effectiveness but less cognitive adverse effects compared to ECT. Moreover, the type and dosage of antipsychotic agents were not restricted, which may confound the results, despite correction with the DDD approach. Furthermore, the various indications for seizure therapy may be another potential confounder; however, the small sample size and multiple indications prevented us from analyzing the effect of different indications on therapeutic efficacy. We failed to investigate the effect of psychiatric comorbidity, e.g., substance abuse or depression, on MST. Considering the comorbidity rate and the fact that depression is the major indication for MST, future research should also address this issue. Compared to patients who received MST, the cognitive assessments of patients who received ECT were closer to the last treatment (though not statistically significant), which may affect their performance. And we only perform one follow-up assessment, which increased the uncertainty of the results. Further studies should consider more time points and higher frequency of cognitive assessment in order to better characterize the overall tolerance along MST course. There were some domains of cognitive functions which is affected by ECT but we did not measure, e.g., autobiographical memory and executive function. In addition, we employed bitemporal electrode placement for ECT with brief pulses, and without individualized dosage titration, which produces larger cognitive side effects than unilateral placement with an ultrabrief pulse and individualized dosage titration (52). The missing data in the cognitive outcomes, though balanced between groups, generated attrition bias. Therefore, the cognitive differences between MST and ECT need confirmation by future studies focusing on the cognitive performance with less attrition, measuring autobiographical memory, executive function, and other relevant cognitive functions. Future studies should confirm the cognitive profiles between MST and the less cognition-affected forms of ECT. Likewise, the parameters of MST, such as coil placement, pulse frequency, and treatment frequency, may also affect the outcomes. Therefore, the optimal parameters for treating schizophrenia should be addressed in future studies.

## Conclusions

The present study revealed that MST and ECT shared similar response rates and antipsychotic patterns among inpatients with schizophrenia. In addition, MST generated fewer cognitive impairments than bitemporal ECT with brief pulses and age-dose method in global cognitive function and several cognitive domains in short term. However, these findings remain to be confirmed by trials with larger sample sizes, more specific indications, less cognition-affected ECT techniques (e.g., ultrabrief ECT with right unilateral or bifrontal placement of electrodes and stimulation titration), and longer follow-up duration. In summary, this interim analysis of an RCT provides preliminary evidence that MST is a promising alternative to ECT as an add-on treatment for schizophrenia.

## Data Availability Statement

The datasets presented in this article are not readily available because the data are only available under the authorization of Shanghai Hospital Development Center. Requests to access the datasets should be directed to https://www.shdc.org.cn/.

## Ethics Statement

The studies involving human participants were reviewed and approved by the Institutional Review Board of the Shanghai Mental Health Center. The patients/participants provided their written informed consent to participate in this study.

## Author Contributions

JL and JJ collected the data. JJ also analyzed the data and drafted the manuscript. BZ and YX were in charge of the randomization. JS, DL, WW, FY, XG, and QL recruited the participants. YJ, TZ, and YT supervised the intervention. JW and CL contributed to the study design and revised the manuscript critically. ZD also revised the manuscript. All the authors approved the final version of the manuscript.

## Funding

This work was supported by grants from the Shanghai Hospital Development Center (SHDC12014111 to CL), the Science and Technology Commission of Shanghai Municipality (13dz2260500 to CL, 14411961400 to JW, and 17411969900 to DL), Shanghai Municipal Commission of Health and Family Planning (201740042 to YJ), National Natural Science Foundation of China (81971251 to JW), and the SHSMU-ION Research Centre for Brain Disorders (to CL). The supporters had no role in the design, analysis, interpretation, or publication of this study.

## Conflict of Interest

The authors declare that the research was conducted in the absence of any commercial or financial relationships that could be construed as a potential conflict of interest.

## Publisher's Note

All claims expressed in this article are solely those of the authors and do not necessarily represent those of their affiliated organizations, or those of the publisher, the editors and the reviewers. Any product that may be evaluated in this article, or claim that may be made by its manufacturer, is not guaranteed or endorsed by the publisher.
