# Supplementary material for: Magnetic Seizure Therapy Compared to Electroconvulsive Therapy for Schizophrenia: A Randomized Controlled Trial
Source: Front Psychiatry. 2021 Nov 25;12:770647. doi: 10.3389/fpsyt.2021.770647 (PMC8656219; doi:10.3389/fpsyt.2021.770647)
Supplement: Supplementary file 1 [file Data_Sheet_1.pdf]

Supplementary materials

Within group analysis of cognitive functions

The within-group analysis of the intention-to-treat set revealed improvement in language function ($t_{(30)} = 2.43$, Bonferroni corrected $p = 0.042$, Hedge's $g = 0.88$, 95% CI = 0.36 to 1.40) among patients who received MST. The within-group analysis also revealed a reduction in immediate memory ($t_{(23)} = -3.61$, Bonferroni corrected $p = 0.003$, Hedge's $g = -1.22$, 95% CI = −2.12 to −0.84) and the RBANS total index ($t_{(23)} = -4.16$, Bonferroni corrected $p = 0.001$, Hedge's $g = -1.71$, 95% CI = −2.37 to −1.04) among patients who received ECT.

The within-group analysis of per protocol set further revealed improvement in the immediate memory ($t_{(24)} = 3.19$, Bonferroni corrected $p = 0.008$, Hedge's $g = 1.28$, 95% CI = 0.67 to 1.89), and language function ($t_{(24)} = 4.76$, Bonferroni corrected $p = 0.000$, Hedge's $g = 1.91$, 95% CI = 1.24 to 2.59) among patients who received MST. The within-group analysis also revealed a reduction in the immediate memory ($t_{(21)} = -3.15$, Bonferroni corrected $p = 0.010$, Hedge's $g = -1.35$, 95% CI = −2.01 to −0.69]), delayed memory ($t_{(21)} = -3.42$, Bonferroni corrected $p = 0.005$, Hedge's $g = -1.47$, 95% CI = −2.13 to −0.80), and the RBANS total index ($t_{(21)} = -3.59$, Bonferroni corrected $p = 0.003$, Hedge's $g = -1.54$, 95% CI = −2.21 to −0.86) among patients who received ECT.

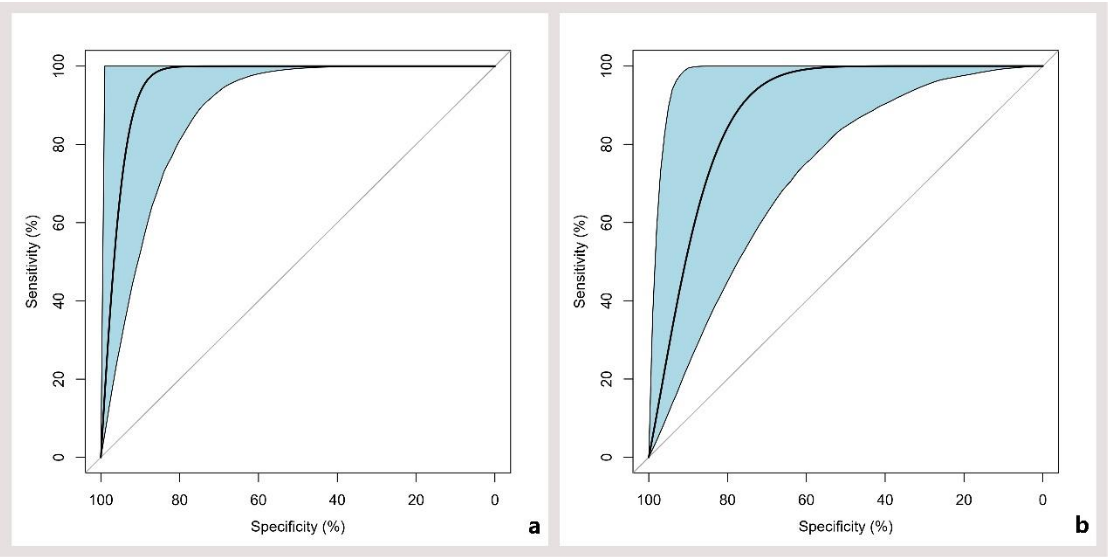

Supplementary Figure 1. Receiver operating characteristic curve for the logistic regression model of predicting intervention response

Clinical response was predicted by **a)** the duration of disease and baseline level of attention for magnetic seizure therapy, with an AUC of 96.3% (95% confidence interval = 90.1% to 99.8%), and by **b)** the baseline level of immediate memory for electroconvulsive therapy, with an AUC of 89.9 % (95% confidence interval = 77.2% to 98.38%).

Estimation of sample size

There was no relevant evidence for response rate of MST for schizophrenia. Therefore, we used the reported response rates in studies comparing ECT and MST in patients with depression [1]. The sample size for each group (n) was calculated using the following formulas [2], with p_A, p_B, α, β, and δ set to 0.4, 0.5, 0.05, 0.2, and 0.1, respectively.

$$n = \left(p_A(1-p_A) + p_B(1-p_B)\right)\left(\frac{(z_{1-\alpha} + z_{1-\beta})}{p_A - p_B - \delta}\right)^2$$

Where

α is Type I error

β is Type II error, meaning $1-\beta$ is power

δ is the testing margin

The estimate was 76, and considering a 10 – 20 % attrition rate, the sample size was eventually set to 90 for each group.

Reference

1. Cretaz E, Brunoni AR, Lafer B. Magnetic Seizure Therapy for Unipolar and Bipolar Depression: A Systematic Review. *Neural Plast.* 2015;2015:521398. doi:10.1155/2015/521398

2. Chow S-C, Shao J, Wang H, Lokhnygina Y. *Sample Size Calculations in Clinical Research.* 2nd Ed. Chapman and Hall/CRC; 2008.
